# Merlin deficiency alters the redox management program in breast cancer

**DOI:** 10.1002/1878-0261.12896

**Published:** 2021-02-01

**Authors:** Mateus Mota, Brandon J. Metge, Dominique C. Hinshaw, Heba A. Alsheikh, Dongquan Chen, Rajeev S. Samant, Lalita A. Shevde

**Affiliations:** ^1^ Department of Pathology University of Alabama at Birmingham AL USA; ^2^ Division of Preventive Medicine University of Alabama at Birmingham AL USA; ^3^ O’Neal Comprehensive Cancer Center University of Alabama at Birmingham AL USA; ^4^ Birmingham VA Medical Center AL USA

**Keywords:** breast, DUOX, merlin, NOX, NRF2, ROS

## Abstract

The expression of Merlin tumor suppressor protein encoded by Neurofibromin 2 (*NF2*) gene is remarkably decreased in metastatic breast cancer tissues. In order to recapitulate clinical evidence, we generated a unique, conditional *Nf2*‐knockout (*Nf2^−/−^*) mouse mammary tumor model. Merlin‐deficient breast tumor cells and *Nf2^−/−^* mouse embryonic fibroblasts (MEFs) displayed a robustly invasive phenotype. Moreover, *Nf2^−/−^* MEFs presented with notable alterations in redox management networks, implicating a role for Merlin in redox homeostasis. This programmatic alteration resonated with pathways that emerged from breast tumor cells engineered for Merlin deficiency. Further investigations revealed that *NF2*‐silenced cells supported reduced activity of the Nuclear factor, erythroid 2 like 2 antioxidant transcription factor, concomitant with elevated expression of NADPH oxidase enzymes. Importantly, mammary‐specific *Nf2^−/−^* in an Mouse mammary tumor virus Neu + murine breast cancer model demonstrated accelerated mammary carcinogenesis *in vivo*. Tumor‐derived primary organoids and cell lines were characteristically invasive with evidence of a dysregulated cellular redox management system. As such, Merlin deficiency programmatically influences redox imbalance that orchestrates malignant attributes of mammary/breast cancer.

## Graphical abstract

Merlin‐deficient cells have an under‐functioning Nuclear factor, erythroid 2 like 2 antioxidant system, as well as a NOX‐enabled redox production system in hyperdrive. This leads to intensified oxidative stress that converges upon unrestricted cell growth and intensification of malignant attributes. Thus, Merlin deficiency disables the cellular redox management system. The findings uncover a novel mechanism by which Merlin intersects with redox homeostasis in restricting malignant characteristics.

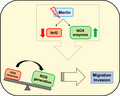

**Abbreviations**
DUOX: Dual oxidase
GCLC: Glutamate‐cysteine ligase catalytic subunit
GCLM: Glutamate‐cysteine ligase modifier subunit
GSH: reduced glutathione
MEF: Mouse embryonic fibroblast
MMTV: Mouse mammary tumor virus
NADPH: Reduced nicotinamide adenine dinucleotide phosphate
NEU: erb‐b2 receptor tyrosine kinase 2
NF2: Neurofibromin 2
NOX: NADPH oxidase
Nrf2: Nuclear factor, erythroid 2 like 2
ROS: Reactive oxygen species
SOD: Superoxide dismutase

## 1 Introduction

The cytoskeleton‐associated Merlin tumor suppressor, encoded by the Neurofibromin 2 (*NF2)* gene, is critically involved in sensing cell–cell contact and bringing about contact‐dependent inhibition of growth. Merlin functions as a scaffold‐like protein that localizes at the cell cortex enabling Merlin to integrate extracellular information to ultimately modulate cellular behavior. *NF2‐*inactivating mutation is a hallmark of Neurofibromatosis type 2 neurological condition. Moreover, low incidence of *NF2* loss‐of‐function mutation also occurs in malignant tumors [1, 2, 3].

We and others have shown that breast cancer is not associated with any significant mutations and transcript‐level alterations in the *NF2* gene [4, 5, 6]. Despite this, we found that Merlin protein levels are significantly reduced in metastatic breast cancer tissues irrespective of the breast cancer subtype. Mechanistically, we demonstrated that Merlin is post‐translationally modified, marking it for ubiquitin‐mediated proteasomal degradation [6]. This frames Merlin as a potentially important regulator of malignant attributes. In order to assess cellular and molecular outcomes of Merlin loss that may contribute to advanced disease, we recapitulated Merlin deficiency in nonmetastatic breast cancer cells. To overcome the challenge of embryonic lethality of a total *Nf2*‐knockout [7], we have generated a unique mammary‐specific *Nf2*‐knockout mouse mammary tumor model. Using an unbiased global transcriptomics approach, we identified that Merlin deficiency programmatically alters a redox management signature, characterized by elevated levels of cellular reactive oxygen species (ROS).

Low‐to‐intermediate levels of ROS can act as second messengers altering protein functions and regulating signaling pathways [8, 9, 10, 11, 12]. In cancer, ROS can reversibly inactivate tumor suppressor proteins, such as phosphatase and tensin homolog [8, 13, 14]; on the other hand, oxidation by ROS can also activate Src family, Ras, and receptor tyrosine kinase (RTK) [11, 12]. Therefore, ROS have become a subject of extensive investigations for supporting tumorigenesis and tumor progression. There is a well‐coordinated antioxidant system to ensure ROS maintenance under a tolerable threshold. Nuclear factor erythroid 2‐related factor 2 (Nrf2) has a key role in the activation of detoxification‐ and antioxidant‐related genes, including those involved in the synthesis and regeneration of reduced glutathione (GSH), the most abundant antioxidant cofactor [15, 16, 17].

In the current study, we have identified that Merlin deficiency disables the redox management system marked by a redox production system in hyperdrive, and an under‐functioning Nrf2 antioxidant system, leading to elevated cellular ROS accumulation. This presents a potentially novel mechanism whereby Merlin keeps ROS levels in check, suppressing tumor formation and progression. This highlights a heretofore unknown mechanism by which Merlin restrains malignant attributes of breast cancer.

## 2 Materials and methods

### 2.1 Human cell lines

MCF7 (ATCC, Manassas, VA, USA) and MCF10AT (Karmanos Cancer Center) cell lines were stably knocked down for *NF2,* generating MCF7 KD and MCF10AT KD, respectively, and cultured as previously described [6, 18]. SUM159 cell lines (Asterand Biosciences, Detroit, MI, USA) were stably restored for *NF2* by transducing pLV‐CMV‐NF2‐GFP‐2A‐Puro lentiviral particles (Capital Biosciences, Gaithersburg, MD, USA) and cultured as previously described [6, 18]. The cell lines T47D and BT474 knocked out for *NF2* were acquired from Synthego, Redwood City, CA, USA and named T47D KD and BT474 KD, respectively. The T47D pair was cultured in RPMI 1640 medium (Thermo Fisher, Grand Island, NY, USA) + 10% heat‐inactivated FBS (Thermo Fisher) + 10 µg·mL^−1^ insulin (Sigma‐Aldrich, St. Louis, MO, USA). The BT474 pair was cultured in RPMI 1640 + 20% heat‐inactivated FBS + 10 µg·mL^−1^ human insulin.

### 2.2 Mouse embryonic cell line generation

*Nf2^fl/fl^* mice (BALB/c) were procured from Riken BioResource Research Center (Tsukuba, Ibaraki, Japan). The strain background was back‐crossed onto pure FVB background in the Animal Facility of the University of Alabama at Birmingham (UAB) in accordance with the guidelines of the IACUC. This was confirmed by genome scanning service by Jackson Laboratory (Bar Harbor, ME, USA). Mouse embryonic fibroblasts (MEFs) were harvested from pregnant *Nf2^fl/fl^* mouse on day 13 (d.13), dissociated into a single‐cell suspension, immortalized with SV40 large T antigen Purified Lentifect Lentiviral Particles (GeneCopoeia, Rockville, MD, USA), and transduced with vector‐control CMV‐GFP lentivirus or CMV‐Cre GFP lentivirus (puro) (Cellomics Technology, Halethorpe, MD, USA), generating MEF *Nf2^fl/fl^* or MEF *Nf2^−/−^,* respectively.

### 2.3 Mouse models

*Nf2^fl/fl^* and mouse mammary tumor virus (MMTV) Neu (FVB/N‐Tg(MMTVneu)202Mul/J, stock no: 002376) mice (Jackson Laboratory) were maintained and crossed to generate *Nf2^fl/fl^* MMTV Neu mice in the Animal Facility of UAB in accordance with the guidelines of the IACUC. This was confirmed by amplification of genomic DNA from tail clips by PCR.

*Nf2^fl/fl^* MMTV Neu mice went through one pregnancy, and litter were weaned 7 days after birth. Following 7 days postweaning, mice were anesthetized in a container with oxygen/isoflurane flow and placed on an illuminated stereoscope with the snout positioned into constant oxygen/isoflurane flow tube. Intraductal injection was performed based on Krause *et al*. method [19]. 3.6 × 10^7^ TU vector‐control CMV‐GFP lentivirus or CMV‐Cre GFP lentivirus (puro) (Cellomics Technology) were intraductally injected into the inguinal mammary glands to generate control *Nf2^fl/fl^* MMTV Neu+ or mammary‐specific *Nf2^−/−^* MMTV Neu+, respectively. The efficiency of the intraductal injection technique was examined by intraductally injecting PBS with 0.2% Evans blue dye, enabling visualization of the mammary ductal tree. Mice were monitored for tumor latency every other day. When tumors reached ~ 12 × 12 mm, mice were euthanized in a CO_2_ chamber and tumor harvested.

### 2.4 RNA sequencing analysis

Total RNA was extracted using the RNeasy Mini Kit (Qiagen, Hilden, Germany) per manufacturer's protocol. Preparation of cDNA libraries, sequencing using next generation sequencing platform, and analysis and interpretation of resultant data were performed by GENEWIZ, South Plainfield, NJ, USA. The datasets used and/or analyzed have been deposited at the Gene Expression Omnibus (GEO) under the accession number GSE157677. Heatmaps for RNA sequencing data were generated using Morpheus (https://software.broadinstitute.org/morpheus). Data were adjusted using one plus log 2, and then, a Marker‐2 selection (*t*‐test) was performed to generate the top and bottom 100 most significantly altered genes. These genes were then run separately through the GSEA Molecular Signature Database (MSigDB) to generate significantly altered upregulated and downregulated pathways [20, 21]. Specifically, GO Pathway signatures (Biological Processes, Molecular Function, and Cell Component) and oncogenic pathway signatures were selected for analysis. Relevant, significantly altered pathways are reported in tables. To generate the network figures, respective RNA‐sequencing datasets were narrowed down to reflect metabolism‐related genes. This was based off of metabolism‐related pathways in the KEGG and PANTHER databases. Metabolism‐relevant genes were analyzed using NetworkAnalyst [22, 23, 24]. Relevant pathways are depicted as network figures with corresponding tables containing relevant gene lists and pathway *P*‐values.

### 2.5 NQO1 luciferase reporter assay

NQO1‐ARE luciferase reporter plasmid was donated by M. Fishel from the Indiana University—School of Medicine. 2 × 10^4^ cells were seeded in 96‐well plates and transfected with 5, 10, or 100 ng of NQO1‐ARE luciferase reporters using Lipofectamine 2000 Transfection Reagent (Invitrogen, Carlsbad, CA, USA) or FuGENE 6 Transfection Reagent (Promega, Madison, WI, USA) (conditions cell line‐dependent). The assay was terminated 30 h post‐transfection and read according to the Luciferase Assay System (Promega) in a GloMax 20/20 luminometer.

### 2.6 Oxidative Stress RT^2^ Profiler PCR Arrays

A panel of oxidative stress‐associated genes was analyzed by an RT^2^ Profiler PCR Array for Human Oxidative Stress or Mouse Oxidative Stress and Antioxidant Defense (Qiagen). Total RNA was harvested as above, genomic DNA was eliminated, and cDNA synthesized according to the RT^2^ First Strand Kit protocol (Qiagen). The array was run on an ABI StepOnePlus (Thermo Fisher) thermocycler using RT^2^ SYBR Green ROX qPCR Mastermix (Qiagen). Data were analyzed in the GeneGlobe Data Analysis Center (Qiagen; https://geneglobe.qiagen.com/us/analyze/). Pro‐oxidant and antioxidant scores were calculated by summing the fold change expression values of selected genes in *NF2*‐silenced or restored cells compared to their controls.

### 2.7 Quantitative RT‐PCR

Total RNA was harvested as above and cDNA synthesized using the High Capacity cDNA Reverse Transcription kit (Applied Biosystems, Vilnius, Lithuania) per manufacturer's protocol. Human and mouse primer probes to query the gene expression of *NF2*, *NOX4*, *DUOX1*, *DUOX2*, *GCLC*, *GCLM*, and *β‐ACTIN* were acquired from Thermo Fisher, and quantitative RT‐PCR was performed with the TaqMan Fast Advanced Master Mix reagent (Applied Biosystems) on an ABI StepOnePlus (Thermo Fisher) thermocycler. Data were analyzed by the 2^−∆∆Ct^ method.

### 2.8 Immunoblotting

Whole cell protein lysates were obtained by lysing cells with RIPA lysis buffer (Millipore, Darmstadt, Germany) supplemented with Halt Protease and Phosphatase Inhibitor Cocktail (Thermo Fisher). Protein lysates were immunoblotted overnight using the following primary antibodies from Cell Signaling Technology (Danvers, MA, USA): Nrf2 (#12721), Keap1 (#8047), Superoxide dismutase (SOD)1 (#4266), Merlin (#12888); Novus Biologicals (Centennial, CO): Nox4 (#NB110‐58849) and Duox2 (#NB110‐61576); and GeneTex (Irvine, CA): Duox1 (#GTX119160). Rabbit IgG HRP‐linked whole antibody (from donkey) and mouse IgG HRP‐linked whole antibody (GE Healthcare, Marlborough, MA) were used as secondary antibodies and β‐actin (Sigma‐Aldrich #A3854) as loading control. Signal was detected by ECL Prime or Select Western blotting Detection Reagents on an Amersham Imager 600 (GE Healthcare). Immunoblot bands were quantified by alpha ease software (Alpha Ease FC, Santa Clara, CA).

### 2.9 ROS assay

ROS levels were assessed by following the instructions of the DCFDA/H2DCFDA‐ Cellular ROS Assay Kit (Abcam, Cambridge, UK) or Cellular ROS assay kit (Deep Red) (Abcam). When applicable, cells were treated with 10 mm l‐Glutathione Reduced (Tocris, Bristol, UK) for 18 h before ROS measurement. Ultra pure water was used vehicle control. Fluorescence was measured by the BD Accuri™ C6 Plus flow cytometer (BD Biosciences, San Jose, CA, USA) using the FITC or APC channels.

### 2.10 Transwell invasion assay

Cells were pretreated with 10 mm l‐Glutathione Reduced (Tocris) or ultra pure water as vehicle control for 18 h, followed by seeding in serum‐free growth medium in BioCoat Matrigel Invasion Chambers (Corning, Bedford, MA, USA). Serum‐free growth medium containing 10 µg·mL^−1^ of fibronectin was added to the bottom of each well where the invasion chambers were inserted. After incubation in 5% CO_2_ incubator at 37 °C for 16 h, cell‐seeded invasion chambers were fixed with 4% paraformaldehyde for 10 min at RT, stained with 0.1% crystal violet/10% EtOH for 10 min at RT, and rinsed with deionized water. Images of four random fields on each of two chambers for each condition were visualized by Nikon Eclipse E200 and photographs of inserts captured using the 10× objective of the DS‐L4 microscope system (Nikon, Tokyo, Japan). Area of invaded cells was measured by imagej (ImageJ.NIH.gov/IJ/Index.html).

### 2.11 3D culture assay

A suspension of 5 × 10^3^ cells in growth media supplemented with 2% 3D Culture Matrix Reduced Growth Factor Basement Membrane Extract (R&D Systems, Minneapolis, MN, USA) was seeded in precoated chamber cover glass slide (Millipore, Ireland) with undiluted 3D Culture Matrix Reduced Growth Factor Basement Membrane Extract (R&D Systems). The seeded cells were incubated in 5% CO_2_ at 37 °C with media replaced every other day; when treated with l‐Glutathione Reduced, media were replaced every day. When acini‐like structures were formed, cells were washed with PBS, fixed with 2% paraformaldehyde + 0.1% glutaraldehyde in PBS for 30 min, permeabilized with 0.1% Triton‐X‐100 for 15 min, and blocked with 5% BSA 0.1% Triton‐X‐100 in PBS for 1 h at room temperature (RT). Cells were labeled with anti‐laminin‐5 (γ2 chain) antibody, clone D4B5 (Millipore #MAB19562) overnight followed by secondary antibody incubation with goat anti‐mouse IgG1 cross‐adsorbed secondary antibody, Alexa Fluor 594 (Invitrogen, Eugene, OR, USA) for 1 h at RT. Nuclei were stained with VECTASHIELD Antifade Mounting Medium with DAPI (Vector Laboratories, Burlingame, CA, USA). Acinar morphology was captured using the 20× objective of Nikon Eclipse Ti‐U microscope (Nikon).

### 2.12 Vinculin/F‐actin immunofluorescence

Cells were seeded on a poly‐l‐lysine‐coated cover slip (Corning). After 48 h under 5% CO_2_ at 37 °C incubation, the cells were fixed with 4% paraformaldehyde for 30 min and permeabilize with 2% Triton‐X‐100 for 15 min at RT. Blocking with 5% BSA + 2% Triton‐X‐100 for 1 h at RT was followed by co‐incubation with Alexa Fluor 594 Phalloidin (Thermo Fisher) and monoclonal anti‐vinculin antibodies (Sigma‐Aldrich) for 1 h at RT. Then, cells were washed 3× with PBS and incubated with goat anti‐mouse IgG (H + L) cross‐adsorbed secondary antibody, Alexa Fluor 488 (Invitrogen, Eugene) for 1 h at RT. After 3× washes with PBS, the cover slip was mounted on a slide with VECTASHIELD antifade mounting medium with DAPI. Images were acquired on Nikon Eclipse Ti‐U using the 40× objective.

### 2.13 Immunohistochemistry

Sections from paraffin‐embedded tissue were deparaffinized and rehydrated through passages in xylene and graded EtOH. Heat‐induced antigen retrieval was performed in boiling sodium citrate buffer for 5 min, followed by incubation with Dual Endogenous Enzyme Block (Dako, Carpinteria, CA, USA) for 15 min. Following 10‐min wash with Tris‐buffered NaCl solution supplemented with 0.01% Triton‐X‐100, tissues were blocked with 3% goat serum for 40 min and incubated with NF2/Merlin Antibody (Novus Biologicals # NBP1‐33531) or HNE‐Michael Adducts (Millipore #393207) overnight in 4 °C. After washing, EnVision + System‐ HRP Labelled Polymer Anti‐Rabbit (Dako) was applied for 40 min at RT. For antibody visualization, tissues were incubated with Liquid DAB + Substrate Chromogen System (Dako) for 7 min and counterstained with Harris Hematoxylin (Surgipath; diluted 1 : 2 in tap water). After tissue dehydration, slides were mounted in Cytoseal (Thermo Scientific) with coverslips. Images were visualized by Nikon Eclipse E200 and captured using the 40× objective of the DS‐L4 microscope system (Nikon). The immunostaining score was based on the method described on Frolova *et al*. [25].

### 2.14 Clinical database

For survival analysis, patient's microarray data for 683 breast cancer primary tumors [26] were accessed from public data portal (https://xenabrowser.net) June 2020. Normalized microarray data were used as NF2 gene expression values, and the median was used to classify samples into high and low expression groups for distant metastasis‐free survival analysis. A Kaplan–Meir curve was generated and log‐rank test applied. Patient's protein data measured by reverse‐phase protein array for 887 breast cancer primary tumors (cohort: Invasive Carcinoma TCGA, Firehouse Legacy) were accessed from public data portal (https://www.cbioportal.org) in August 2020. Data were extracted for analysis, and NF2 protein expression was examined in correlation of Neoplasm Disease Lymph Node Stage American Joint Committee on Cancer Code. Another cohort of 747 breast cancer primary tumors from TCGA Breast Cancer—BRCA was accessed from public data portal (https://xenabrowser.net) in August 2020. Data were extracted for analysis, and NF2 protein expression was examined in correlation of axillary lymph node stage (Pathological_N). One‐way ANOVA and Tukey *post hoc* tests were applied for statistical analysis, using graphpad prism version 8 (GraphPad Software, La Jolla, CA, USA). Comparisons were considered statistically significant for *P*‐value < 0.05.

### 2.15 Statistical analysis

An unpaired Student's *t*‐test was applied for statistical analysis, unless otherwise mentioned, using graphpad prism version 8. Comparisons were considered statistically significant for *P*‐value < 0.05. Error bars represent ± SEM.

## 3 Results

### 3.1 Merlin deficiency modulates a redox signaling signature

**Fig. 1:**
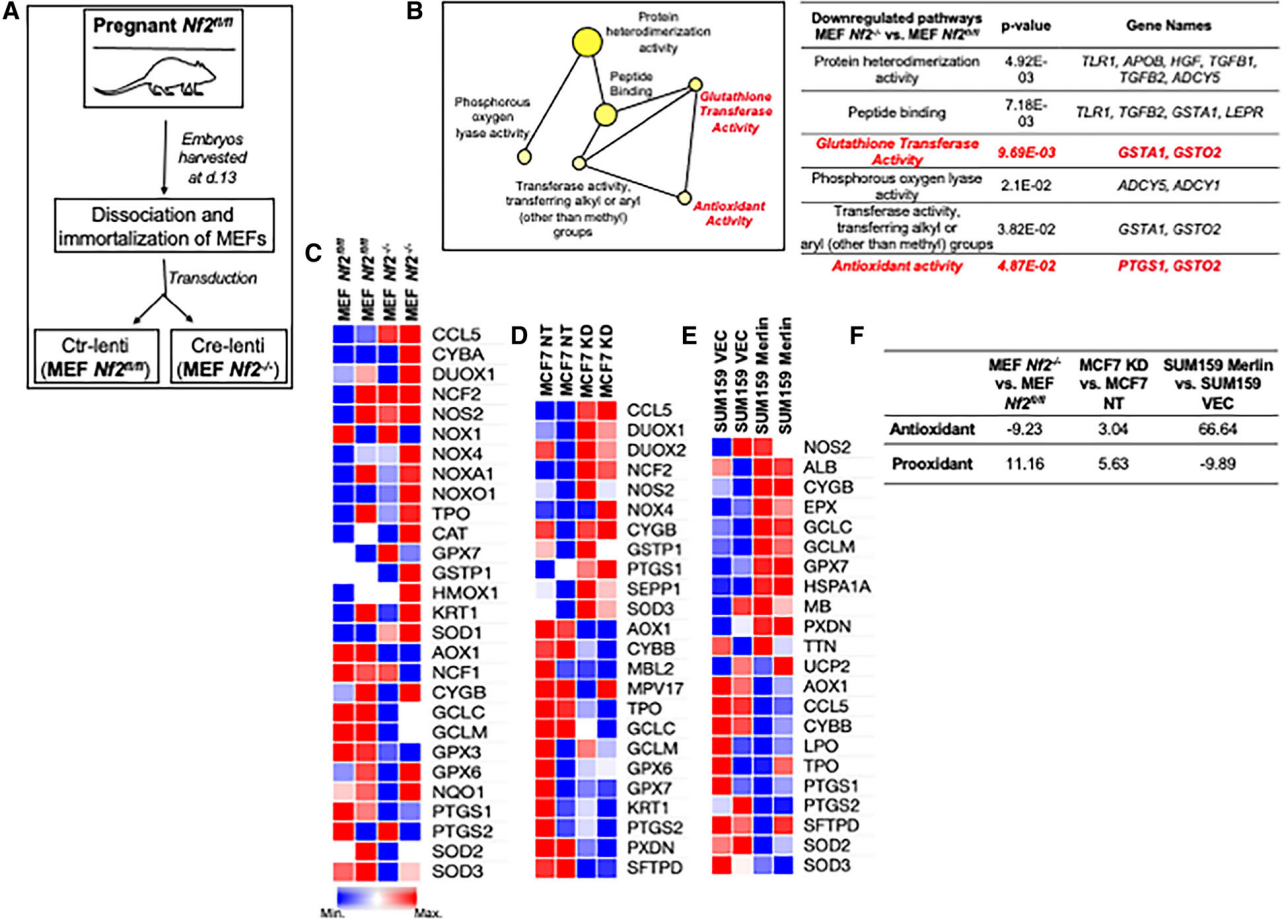
Merlin deficiency modulates a redox signaling signature. (A) MEFs were harvested from pregnant *Nf2^fl/fl^* mouse on day 13 (d.13), dissociated into single‐cell suspension, immortalized with SV40 large T antigen lentiviral particles, and transduced with vector‐control lentivirus (Ctr‐lenti) or Cre‐lentivirus (Cre‐lenti), generating MEF *Nf2^fl/fl^* or MEF *Nf2^−/−^,* respectively. (B) Glutathione transferase and antioxidant‐associated genes were downregulated in MEF *Nf2^−/−^* compared to MEF *Nf2^fl/fl^* (*n* = 3 for each group). Student's *t*‐test was applied for statistical analysis. A panel of redox‐associated genes was assessed by gene expression array, and selected genes were plotted as heatmap (*n* = 2 for each group) for (C) MEF *Nf2^fl/fl^* and MEF *Nf2^−/−^*, (D) control and *NF2*‐silenced MCF7 (MCF7 NT and MCF7 KD, respectively), and (E) control and *NF2*‐restored SUM159 (SUM159 VEC and SUM159 Merlin, respectively). (F) Antioxidant and pro‐oxidant score of MEF *Nf2^−/−^* x MEF *Nf2^fl/fl^*, MCF7 KD x MCF7 NT, and SUM159 Merlin x SUM159 VEC.

In order to evaluate a role for Merlin in the context of normal biology, we harvested embryonic fibroblasts from *Nf2^fl/fl^* female mice and immortalized these MEFs with SV40 large T antigen lentiviral particles. We stably transduced these MEFs with either Cre‐expressing lentivirus to knockout *Nf2 (*MEF *Nf2^−/−^)* or control empty vector (MEF *Nf2^fl/fl^*) (Fig. 1A). In order to characterize alterations in gene expression as a result of *Nf2* loss, we sequenced the transcriptome of these MEFs (Fig. S1A). This revealed prominent alterations in metabolism‐related pathways. Further data inquiry through the Network Analyst platform revealed significant downregulation of signatures related to glutathione transferase and antioxidant activities (Fig. 1B). We also performed RNAseq analysis of MCF10AT breast tumor cells stably silenced for *NF2* (MCF10AT KD). Similar to *Nf2^−/−^* MEFS, MCF10AT cells deficient for Merlin showed downregulation of redox‐associated pathways compared to their control MCF10AT NT cells (Fig. S1B). These data suggest that the association between Merlin and redox mechanisms is not breast cancer system‐specific; rather it is a direct consequence of Merlin deficiency.

Redox imbalance is reflective of either decreased clearance or increased production of ROS. Thus, in order to examine the effect of Merlin deficiency in modulating the cellular redox profile, we assessed a panel of human oxidative stress‐associated genes by a quantitative PCR array comparing *NF2*‐silenced breast cancer cells MCF7 (MCF7 KD), T47D (T47D KD), and MEF *Nf2^−/−^* to their respective non‐target control transfectants MCF7 (MCF7 NT) (Fig. S1C), T47D (T47D NT) (Fig. S1D), and MEF *Nf2^fl/fl^* (Fig. S1E). Cells stably silenced for Merlin show significant alterations in antioxidant and pro‐oxidant genes [MEFs (Fig. 1C) and MCF7 (Fig. 1D)]. Moreover, silencing of *NF2* manifests as a lower magnitude of change in antioxidant genes relative to pro‐oxidant genes (Fig. 1F). Conversely, the redox profile of *NF2‐*restored SUM159 (SUM159 Merlin) and its vector control SUM159 (SUM159 VEC) (Figs 1E and S1F) showed higher magnitude of cumulative change of antioxidant genes than pro‐oxidant genes (Fig. 1F). Collectively, the data support a role for Merlin in regulating redox homeostasis in breast cancer cells.

### 3.2 ROS clearance attenuates migratory and invasive phenotypes in *NF2*‐deficient cells

**Fig. 2:**
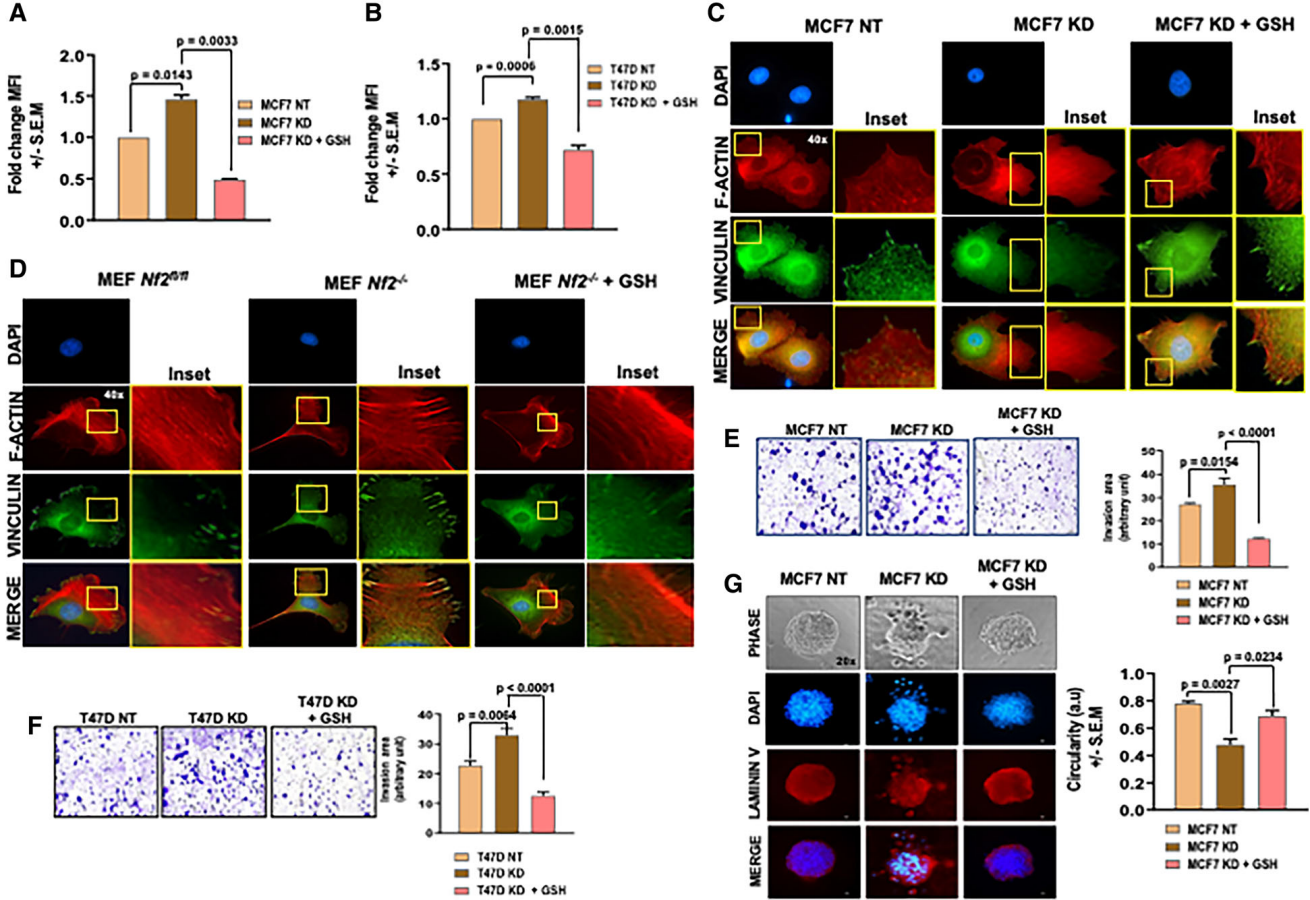
ROS clearance attenuates migratory and invasion phenotypes in *NF2*‐deficient cells. (A) MCF7 KD and (B) T47D KD cells show significantly elevated ROS levels than MCF7 NT (*P* = 0.0143) and T47D NT (*P* = 0.0006), respectively, and exogenous GSH treatment (+ GSH) reduced intracellular ROS levels in both MCF7 KD (*P* = 0.0033) and T47D KD (*P* = 0.0015) cells (*n* = 2 for each group). (C) MCF7 KD and (D) MEF *Nf2^−/−^* displayed longer and more extended lamellipodia with disorganized arrangement of actin filaments (inset) than MCF7 NT and MEF *Nf2^flfl^*, respectively; this phenotype was attenuated by +GSH. Scale bar = 50 µm. Images representative of at least three different fields of each group. Transwell invasion assay showed enhanced invasion ability of (E) MCF7 KD and (F) T47D KD compared to MCF7 NT cells (*P* = 0.0154) and T47D NT (*P* = 0.0064), respectively, and + GSH reduced invasion of both MCF7 KD (*P* < 0.0001) and T47D KD (*P* < 0.0001) cells; quantification of invasion was calculated based on images of four different fields of each group (*n* = 2) and represented as invasion area (arbitrary units). Scale bar = 100 µm. (G) MCF7 KD cells presented a breached basement membrane in contrast to an intact, circumscribed one of MCF7 NT cells when analyzed by laminin V‐stained 3D cell culture; + GSH attenuated the invasion behavior of MCF7 KD cells. Circularity of spheroids (arbitrary units) was measured based on three different 3D‐culture organoids (MCF7 KD x MCF7 NT; *P* = 0.0027) (MCF7 KD + GSH × MCF7 KD; *P* = 0.0234). DAPI was used to label nuclear DNA. Scale bar = 50 µm. Error bars represent ± SEM. Student's *t*‐test was applied for statistical analysis.

Based on leads presented thus far, we surmised that loss of Merlin would alter cellular levels of ROS. In fact, Merlin‐deficient MCF7 and T47D cells show significantly greater levels of total ROS (Fig. 2A,B). In order to test if higher ROS accumulation could be a consequence of defective quenching mechanisms, we cultured cells in medium supplemented with GSH (+ GSH). Exogenous administration of cell‐permeable GSH reduced ROS levels in both, MCF7 KD (Fig. 2A) and T47D KD (Fig. 2B) breast cancer cells, suggestive of a dysfunctional ROS clearance system in context of Merlin deficiency.

As a tumor suppressor recognized for its role in regulating proliferation, we queried the relationship between deficiency of Merlin protein and clinical attributes of breast cancer using publicly available data. Tumor tissues deficient in Merlin protein expression show greater lymph node involvement as coded by nodal status assessment from different studies (Fig. S2A,B). The ability of tumor cells to spread and invade is a key behavior associated with tumor progression and aggressiveness. Interestingly, MEF *Nf2^−/−^* cells showed an upregulated molecular signature associated with cytoskeletal rearrangement and cell motility compared to MEF *Nf2^fl/fl^* cells (Fig. S2C). We assessed focal adhesion, an important attribute of cell migration, by staining for vinculin/F‐actin assembly. Vinculin is essential at focal adhesions, providing the mechanical force for traction and strengthening of integrin‐F‐actin linkages. While MCF7 NT (Fig. 2C) and MEF *Nf2^fl/fl^* (Fig. 2D) presented with shorter and less protruded lamellipodia, MCF7 KD (Fig. 2C) and MEF *Nf2^−/−^* (Fig. 2D) displayed longer and more extended lamellipodia, suggesting enhanced migratory capability. In addition, actin filaments are less organized in the MCF7 KD (Fig. 2C‐inset) and MEF *Nf2^−/−^* (Fig. 2D‐inset) cells compared to their controls, reaffirming the role of Merlin in arranging the cytoskeleton. Exogenous GSH reversed these cytoskeletal changes suggesting that elevated ROS levels impinge upon focal adhesions in the context of Merlin deficiency (Fig. 2C,D). Concordant with this, MCF7 KD and T47D KD cells are remarkably more invasive than MCF7 NT (Fig. 2E) and T47D NT cells (Fig. 2F), respectively, and GSH mitigates this invasiveness in KD cells. In order to evaluate the invasive properties in 3D, we cultured cells as spheroids in a 3D matrix. MCF7 NT spheroids displayed an intact basement membrane stained with laminin V and formed a circumscribed acinar structure in 3D cell culture (Fig. 2G). On the other hand, MCF7 KD spheroids presented a breached basement membrane, with distinct projections indicating an invasive phenotype (Fig. 2G). Treatment of MCF7 KD cells with exogenous GSH attenuated this invasive behavior, indicating that elevated ROS levels contribute to notably enhanced malignant phenotypes in *NF2*/Merlin‐deficient cells. Thus, in the context of Merlin deficiency, elevated ROS configures an invasive phenotype.

### 3.3 Merlin‐deficient breast cancer cells display a dysfunctional antioxidant system

**Fig. 3:**
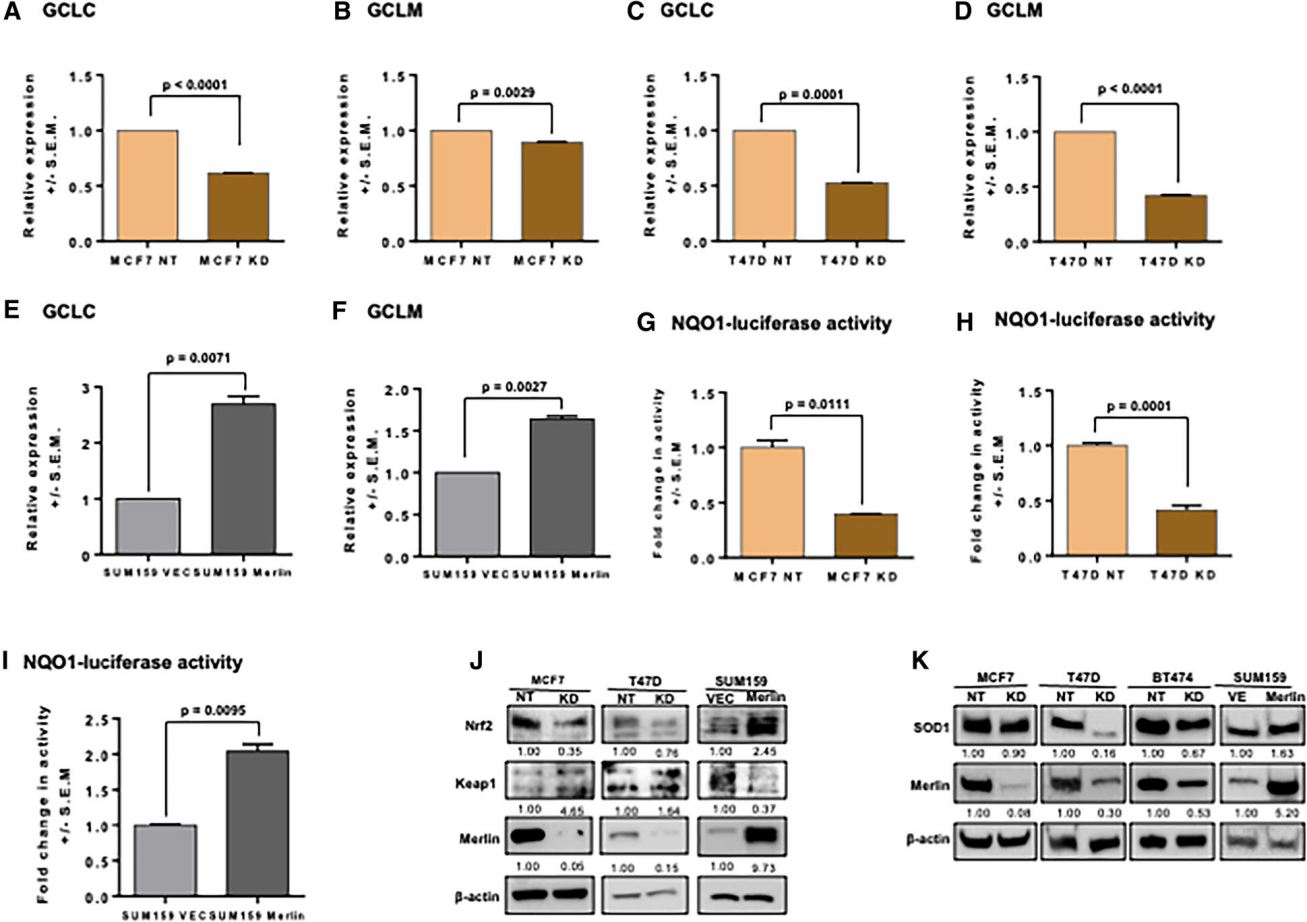
Merlin‐deficient breast cancer cells display a dysfunctional antioxidant system. Expression levels of *GCLC* and *GCLM* were decreased in MCF7 KD (A and B, respectively) (*P* < 0.0001 and *P* = 0.0029, respectively) and T47D KD (C and D, respectively) (*P* = 0.0001 and *P* < 0.0001, respectively) compared to their NT controls. In contrast, expression levels of *GCLC* and *GCLM* were increased in SUM159 Merlin (E and F, respectively) (*P* = 0.0071 and *P* = 0.0027, respectively) compared to SUM159 VEC. Nrf2 activity was measured by an NQO1‐driven Nrf2 luciferase assay which showed decreased activity in MCF7 KD (G) (*P* = 0.0111) and T47D KD (H) (*P* = 0.0001) compared to their respective NT controls; Nrf2 activity was increased in (I) SUM159 Merlin (*P* = 0.0095) compared to SUM159 VEC (*n* = 3 for each group). (J) Protein levels of Nrf2 were decreased while Keap1 increased in Merlin‐deficient cells compared to controls; the opposite was seen in the comparison between SUM159 Merlin and SUM159 VEC (*n* = 3 for each group). (K) SOD1 protein expression is decreased in *NF2*‐silenced breast cancer cells, MCF7 KD, T47D KD, and BT474 KD compared to their non‐target (NT) controls. In contrast, SOD1 is upregulated in SUM159 Merlin compared to SUM159 VEC. Error bars represent ± SEM. Student's *t*‐test was applied for statistical analysis. Band densitometry of immunoblotting is shown. β‐actin was used as loading control.

GSH is synthesized *de novo* in a two‐step reaction where the rate‐limiting step is mediated by glutamate‐cysteine ligase (GCL). GCL is comprised of the GCL catalytic (*GCLC*) and GCL modifier (*GCLM*) subunits [27, 28]. Expression of these two subunits was negatively impacted by Merlin deficiency (Fig. 1C,D) while restoring Merlin induced the expression of both genes (Fig 1E). Further, validation of *GCLC* and *GCLM* transcript levels showed that their steady‐state expression was downregulated in MCF7 KD (Fig. 3A,B, respectively), T47D KD (Fig. 3C,D, respectively), and MCF10AT KD (Fig. S3A,B, respectively) compared to their NT controls. Conversely, gene expression of both, *GCLC* and *GCLM*, was upregulated in SUM159 Merlin expressors (Fig. 3E,F) compared to SUM159 VEC, suggesting that Merlin impacts key determinants of the rate of GSH *de novo* synthesis.

Both, *GCLC* and *GCLM* are *bonafide* transcriptional targets of the Nrf2 transcription factor. Nrf2 is a master regulator in cellular stress response and plays a key role in the activation of antioxidant and detoxification genes. We used an NQO1‐driven Nrf2 luciferase assay as a readout of Nrf2 activity [29]. Nrf2 activity was significantly reduced in MCF7 KD (Fig. 3G), T47D KD (Fig. 3H), and MCF10AT KD (Fig. S3C) cells compared to their respective controls; while SUM159 Merlin expressor cells supported upregulated Nrf2 activity (Fig. 3I). The protein levels of Nrf2 were aligned with the levels of Merlin (Figs 3J and S3D). Nrf2 is a substrate of Keap1 adaptor protein which assists in Nrf2 degradation via Cul3‐dependent ubiquitin ligase complex. *NF2*‐silenced cells showed elevated Keap1 expression compared to their controls (Fig. 3J) suggesting that attenuation of Nrf2 activity in conditions of Merlin deficiency may be caused by increased Keap1‐dependent Nrf2 degradation.

We see that the levels of SOD1, also an Nrf2 transcriptional target [30], are decreased in breast tumor cells deficient for Merlin in comparison to their respective controls; while SUM159 Merlin expressors support increased SOD1 levels (Fig. 3K). SOD1 mediates the conversion of superoxide ($O_{2}^{\cdot ‐}$) into hydrogen peroxide (H_2_O_2_) so it can be broken down into H_2_O in a GSH‐dependent enzymatic reaction. Decreased SOD1 levels also foster conditions that are permissive for accumulation of superoxide ($O_{2}^{\cdot ‐}$) in cells [31]. As such, reduced Nrf2 activity, decreased levels of *GCLC*, *GCLM*, and SOD1 cumulatively reflect a remarkable decrease in the antioxidant system, corresponding with an overall increase in cellular ROS in Merlin‐deficient cells.

### 3.4 Merlin deficiency upregulates proteins from the pro‐oxidative NOX family

**Fig. 4:**
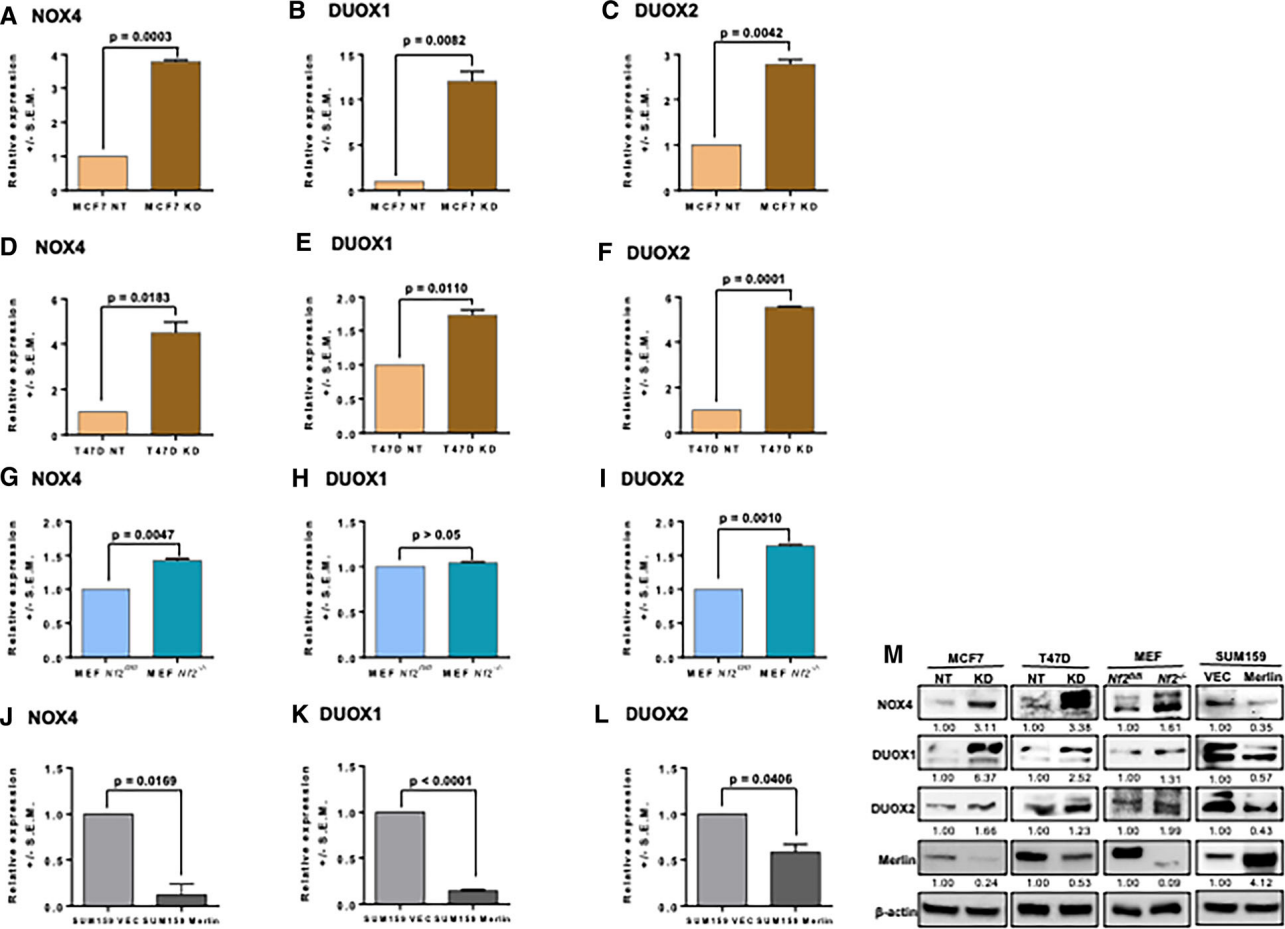
Merlin deficiency upregulates proteins from the pro‐oxidative NOX family. Gene expression of NOX4, DUOX1, and DUOX2 was upregulated in MCF7 KD (A) (*P* = 0.0003), (B) (*P* = 0.0082), (C) (*P* = 0.0042), respectively, and T47D KD (D) (*P* = 0.0183), (E) (*P* = 0.0110), (F) (*P* = 0.0001), respectively, compared to their NT controls. Expression of (G) NOX4 (*P* = 0.0047) was upregulated in MEF *Nf2^−/−^* compared to MEF *Nf2^fl/fl^*; however, no significant expression change was observed for DUOX1 (H) (*P* > 0.05). In contrast, DUOX2 (I) (*P* = 0.0010) was upregulated in MEF *Nf2^−/−^* compared to MEF *Nf2^fl/fl^*. Gene expression of NOX4 (J) (*P* = 0.0169), DUOX1 (K) (*P* < 0.0001), and DUOX2 (L) (*P* = 0.0406) was downregulated in SUM159 Merlin compared to SUM159 VEC (*n* = 3 for each group). (M) Validation of the levels of NOX4, DUOX1, and DUOX2 was assessed by immunoblotting. Error bars represent ± SEM. Student's *t*‐test was applied for statistical analysis. β‐actin was used as loading control. Representative immunoblot band densitometry is shown.

In addition to a compromised antioxidant system, increased ROS accumulation could result from increased ROS generation. Among the pro‐oxidant‐associated genes detected in the gene expression array of Merlin‐deficient cells (Fig. 1C,D), NADPH oxidase 4 (NOX4), Dual oxidase 1 (DUOX1) and DUOX2 were of great interest because they belong to the NOX family which is recognized for its role in generating ROS. Thus, in order to confirm the impact of Merlin deficiency on the expression of NADPH oxidase enzymes, we evaluated the steady‐state transcript levels of NOX4, DUOX1, and DUOX2. Expression of all—NOX4, DUOX1, DUOX2—was upregulated in MCF7 KD (Fig. 4A–C) and T47D KD (Fig. 4D–F) cells compared to their control counterparts. Additionally, expression of NOX4 and DUOX2 was also increased in MCF10AT KD compared to NT cells (Fig. S4). Not just limited to tumor cells, we see that MEF *Nf2^−/−^* cells support elevated levels of NOX4 and DUOX2 (Fig. 4G–I) compared to MEF *Nf2^fl/fl^*. In contrast, SUM159 cells restored for Merlin showed a significant decrease in NOX4, DUOX1, and DUOX2 transcripts (Fig. 4J–L). The expression of NOX4, DUOX1, and DUOX2 proteins is largely consistent with the observed transcript levels (Fig. 4M). Overall, we see that expression of NADPH oxidase enzymes is increased in Merlin‐deficient conditions and re‐expression of Merlin reverses this trend.

### 3.5 Genetically engineered oncogene‐driven Merlin‐deficient mammary tumors harbor elevated oxidative stress

**Fig. 5:**
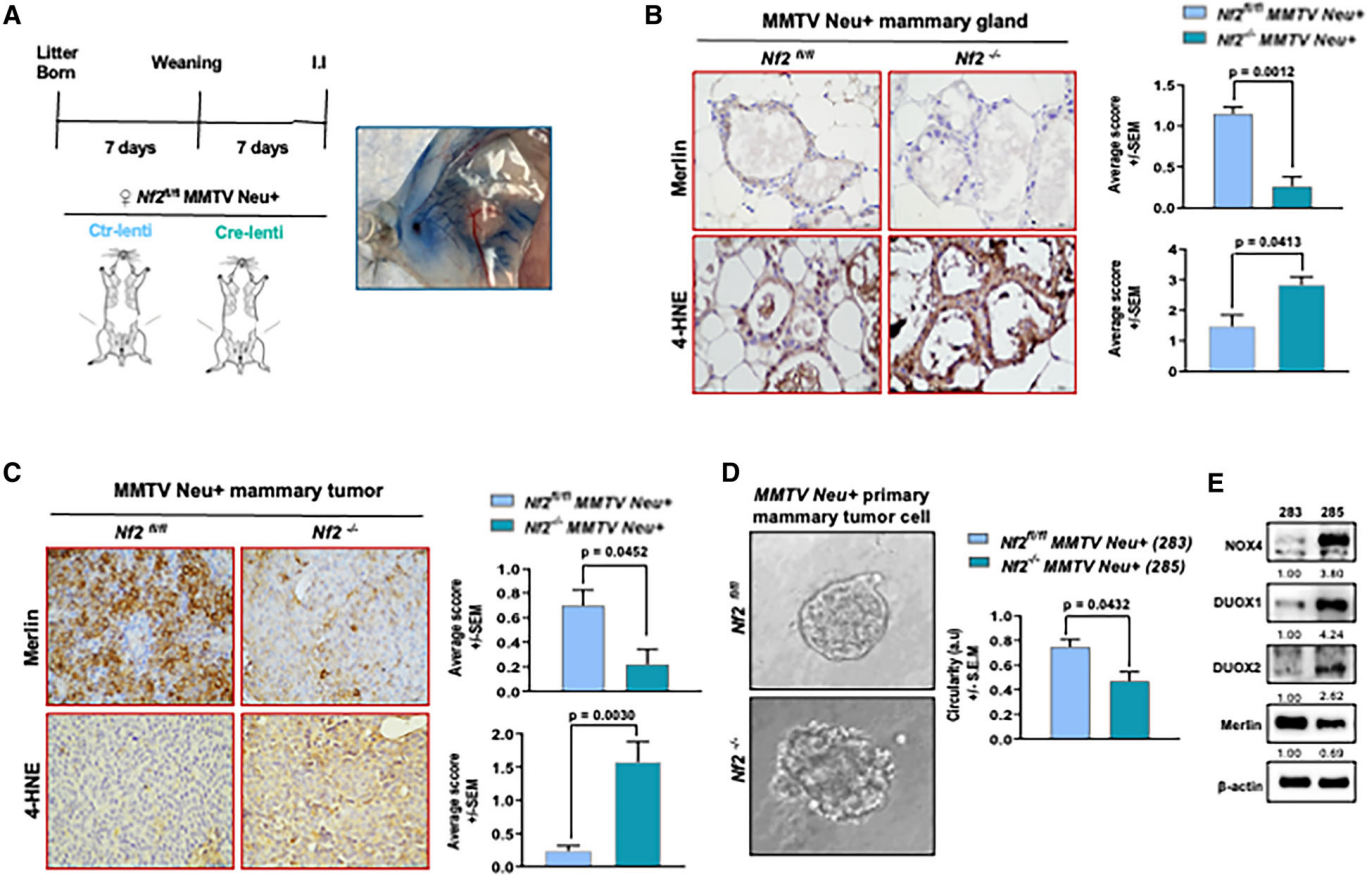
Genetically engineered oncogene‐driven Merlin‐deficient mammary tumors harbor elevated oxidative stress. (A) Pups of pregnant *Nf2^flfl^* MMTV Neu mice were weaned 7 days after birth, and the mice were intraductally injected (I.I) with vector‐control lentivirus (Ctr‐lenti) or Cre‐lentivirus (Cre‐lenti) in both 4th mammary glands 7 days postweaning to generate *Nf2^flfl^* MMTV Neu+ and *Nf2^−l−^* MMTV Neu + mice, respectively. I.I efficiency test with PBS with 0.2% Evans blue dye enabling the visualization of the ductal tree is shown. (B) Loss of Merlin and increased 4‐HNE staining are observed in mammary gland harvested from *Nf2^−l−^* MMTV Neu + compared to *Nf2^flfl^* MMTV Neu + by IHC; IHC score for Merlin (*P* = 0.0012) and 4‐HNE (*P* = 0.0413) is shown. Scale bar = 30 μm. Images representative of 4 different fields of each group (*n* = 2). (C) Mammary tumor tissue harvested from *Nf2^−l−^* MMTV Neu + showed lower levels of Merlin and increased 4‐HNE staining than *Nf2^flfl^* MMTV Neu+; IHC score for Merlin (*P* = 0.0452) and 4‐HNE (*P* = 0.0030) is shown. Scale bar = 30 μm. Images representative of 4 different fields of each group (*n* = 2). (D) 3D cell culture of primary mammary tumor cells harvested from *Nf2^−l−^* MMTV Neu + showed a very breached and disorganized structure compared to *Nf2^flfl^* MMTV Neu+. Circularity of the spheres is shown (*P* = 0.0432). Scale bar = 50 µm. Images representative of three different 3D‐culture organoids. (E) *Nf2^flfl^* MMTV Neu+‐and *Nf2^−l−^* MMTV Neu+‐derived primary tumor cells were immortalized and named 283 and 285, respectively; 285 cells had lower levels of Merlin and higher levels of NOX4, DUOX1, and DUOX2 than 283. Error bars represent ± SEM. Student's *t*‐test was applied for statistical analysis. β‐actin was used as loading control. Representative immunoblot band densitometry is shown.

In order to investigate the functional and mechanistic role of Merlin on mammary tumor development *in vivo*, we generated a *Nf2^fl/fl^* MMTV Neu mouse model and intraductally injected female mice with Cre‐expressing lentivirus to generate a mammary‐specific *Nf2* knockout mouse (*Nf2^−/−^* MMTV Neu+) (Fig. 5A). We confirmed *Nf2* deletion in the mammary gland by immunohistochemistry (IHC) that showed significantly reduced Merlin levels in *Nf2^−/−^* MMTV Neu + mammary glands compared to *Nf2^fl/fl^* MMTV Neu + mammary glands (Fig. 5B). The minimum residual amounts of Merlin in the *Nf2‐*deleted tissue are likely a consequence of mosaicism of Cre activity. 4‐hydroxynonenal (4‐HNE) serves as a surrogate indicator of oxidative stress in tissues [32, 33]. We stained these mammary glands for 4‐HNE. *Nf2^−/−^* MMTV Neu + mammary glands demonstrated remarkably increased staining for 4‐HNE residues compared to glands from *Nf2^fl/fl^* MMTV Neu + mice (Fig. 5B), recapitulating the *in vitro* impact of Merlin deficiency in the accumulation of ROS.

In the *Nf2*‐deleted mammary glands, tumor latency was shorter ‐ at day 56, 50% of the mice in the *Nf2^−/−^* MMTV Neu + group were tumor‐free, while that mark was hit at day 74 for the control group (Fig. S5A). In addition, the level of Merlin in *Nf2^−/−^* MMTV Neu+‐derived tumor tissue was significantly lower than in the tumors derived from *Nf2^fl/fl^* MMTV Neu + mice (Fig. 5C), suggesting that faster tumor onset was attributable to *Nf2* deletion. In order to score the level of oxidative stress in the tumor tissues, we evaluated 4‐HNE staining. The intensity of 4‐HNE detection was significantly greater in *Nf2^−/−^* MMTV Neu+‐derived tumor tissue (Fig. 5C), indicative of an increased oxidative stress in these tumors that developed with shorter latency.

In order to evaluate the phenotype that these tumor cells would display in 3D, we evaluated the morphology of tumor‐derived organoids in 3D culture. *Nf2^fl/fl^* MMTV Neu+‐derived primary tumor cells formed spheroids with a defined, intact morphology and few projections. This was in stark contrast with *Nf2^−/−^* MMTV Neu + organoids that presented a breached, disorganized structure (Fig. 5D). We also established primary cell lines from *Nf2^fl/fl^* MMTV Neu+‐ (283) and *Nf2^−/−^* MMTV Neu+‐ (285) derived primary tumors. Interestingly, 285 showed notably higher levels of NOX4, DUOX1, and DUOX2 than 283 (Fig. 5E), further supporting that the expression of these pro‐oxidative proteins is regulated by Merlin.

## 4 Discussion

The role of ROS in cancer biology has been contentious. Basal levels of ROS are critical to relay signal transduction and maintain proper tissue function [34, 35]. However, distorted redox homeostasis during tumorigenesis as well as metastasis can be lethal. In order to avoid oxidative stress, tumor cells increase their antioxidant ability to prevent toxic accumulation of ROS [28].

Breast cancer patients with low‐Merlin expressing tumors manifested higher rates of distant metastasis‐free survival compared to patients with tumors having higher levels of Merlin (Fig. S5B). Our investigations have revealed that Merlin deficiency dysregulates the cellular redox management program, eliciting a markedly invasive phenotype in MEFs and breast cancer cells. This is also recapitulated in our mammary‐specific *Nf2*‐knockout mice, which presented with significantly accelerated development of invasive tumors.

Participating as a cofactor in antioxidant enzymatic reactions, GSH enables breakdown of H_2_O_2_ into nontoxic components [27, 36]. In Merlin‐deficient model systems, dysregulated ROS accumulation impacted an invasive cytoskeletal reconfiguration. Exogenous GSH mitigated the effects of Merlin deficiency, evident as decreased invasive potential of *NF2/*Merlin‐defective cells. These data established a role for elevated ROS in regulating malignant behavior in the context of Merlin deficiency and put the spotlight on the Nrf2 transcription factor.

Nrf2 homeostatic levels are regulated by ubiquitination and degradation mediated by KEAP1, an E3 ubiquitin ligase substrate adaptor. Under oxidative stress, KEAP1 is oxidized and undergoes a conformational change, releasing its repression on Nrf2 [16, 17, 37]. Breast tumor cells engineered for Merlin deficiency supported significantly diminished Nrf2 activity, marked by decreased levels of antioxidant regulators and effectors. Additionally, when Merlin levels are compromised, NOX4, DUOX1, and DUOX2 proteins were significantly elevated. These NOX enzymes actively generate ROS [34, 38, 39] and are upregulated in several types of malignancies including breast cancer [6, 40, 41, 42, 43, 44, 45, 46, 47]. Notably, oxidative stress resultant of NOX‐derived ROS is the main promoter of tumorigenesis, invasion and metastasis [9]. Our investigations have revealed that Merlin deficiency disables the redox management system. The redox production system is aberrantly activated simultaneous with an under‐functioning of the Nrf2 antioxidant system, leading to intensified oxidative stress. Oxidative stress aids in the formation of invadopodia during tumor cell invasion and migration by enabling cytoskeletal rearrangements, and by stimulating proteolytic degradation of ECM components [48, 49]. In a breast cancer model, tumor cells that survived hypoxia showed a ROS‐resistant phenotype that provided invasive and metastatic advantage [50]. We found that Merlin‐deficient cells are unable to effectively limit ROS accumulation, and also are resistant to and survive the resultant oxidative stress, with a remarkable invasive phenotype.

White *et al*. demonstrated that in cells harboring mutant Merlin, activation of YAP/TAZ programs a metabolic circuitry that enables growth and proliferation. In the absence of exogenous growth factors, *NF2*‐mutant tumor cells rely on YAP/TAZ‐signaling to maintain growth‐promoting signaling through engaging the activities of AKT and RTKs. Interestingly, they reported that YAP/TAZ function to limit mitochondrial respiration, prevent ROS buildup, and reduce oxidative stress cell death under nutrient stress such as glutamine deprivation [51]. We previously demonstrated that Merlin‐deficient breast cancer cells metabolically adapt toward aerobic glycolysis by co‐operatively engaging SMAD‐Hippo signaling [52]. Our current findings indicate that Merlin deficiency enables ROS accumulation—a contrast to the previous study that can be explained by several factors—our studies are centered on breast cancer cells that do not harbor mutations in *NF2* and are not conducted in nutrient‐limiting conditions. Additionally, NOX enzymes are well‐known producers of intracellular ROS. This suggests that the main source of generation of ROS in our system is not sustained by mitochondrial‐related activities. It also is likely that co‐operative activity of Hippo and SMAD signaling orchestrates a metabolism in nutrient‐replete conditions that is distinct from that in nutrient‐limiting conditions.

In the cell cortex, Merlin integrates extracellular cues and intracellular responses [1, 2, 3]. As such, its loss or deficiency enables the activation of several signaling pathways that converge upon unrestricted cell growth [53]. Many of these mechanisms can lead to elevated cellular ROS, which in turn, can aberrantly activate several signaling nodes [9, 54, 55]. Thus, it is unsurprising that a total Merlin knockout has an embryonic lethal phenotype. Our new mammary model of Merlin deficiency surmounts this limitation and uncovers a potential mechanism whereby Merlin keeps levels of ROS in check, suppressing mammary tumor formation and invasive behavior. The findings endorse a novel mechanism whereby Merlin restrains tumorigenesis and tumor progression.

## 5 Conclusion

In conclusion, we find that lack of normal Merlin function induces mismanagement of the cellular redox system, converging upon unrestricted cell growth and intensification of malignant attributes. Mechanisms of ROS management, represented by Nrf2‐related clearance functions and ROS generation associated with NADPH oxidase enzyme family, were altered in Merlin‐deficient breast cancer cells. This is also reflected in elevated levels of oxidative stress marked by 4‐HNE staining. The use of MEFs, a noncancerous cell system, evidences that the impact of Merlin deficiency reaches beyond breast cancer biology. The findings uncover a novel mechanism by which Merlin intersects with redox homeostasis in restricting malignant characteristics.

## Conflict of interest

The authors declare no conflict of interest.

## Author contributions

MM, BM, RS, and LS designed the study and experiments. MM conducted the *in vitro* experiments. MM and BM conducted the *in vivo* experiments. DH acquired the MSigDB. HA acquired the clinical database. DC extracted and edited the RNA sequencing dataset. MM, BM, DH, HA, DC, RS, and LS analyzed and interpreted the results. MM and LS wrote the manuscript.

## Supporting information


**Fig. S1.** Merlin deficiency modulates a redox signaling signature.

**Fig. S2.** ROS clearance attenuates migratory and invasion phenotypes in *NF2*‐deficient cells.

**Fig. S3.** Merlin‐deficient breast cancer cells display a dysfunctional antioxidant system.

**Fig. S4.** Merlin deficiency upregulates proteins from the pro‐oxidative NOX family.

**Fig. S5.** Genetically engineered oncogene‐driven Merlin‐deficient mammary tumors harbor elevated oxidative stress.

## Data Availability

The datasets for the RNA sequencing of the MEF cell lines have been deposited at the GEO under the accession number GSE157677.
