# Supplementary figures and images for: Merlin deficiency alters the redox management program in breast cancer
Source: Mol Oncol. 2021 Feb 1;15(4):942–56. doi: 10.1002/1878-0261.12896 (PMC8024723; doi:10.1002/1878-0261.12896)

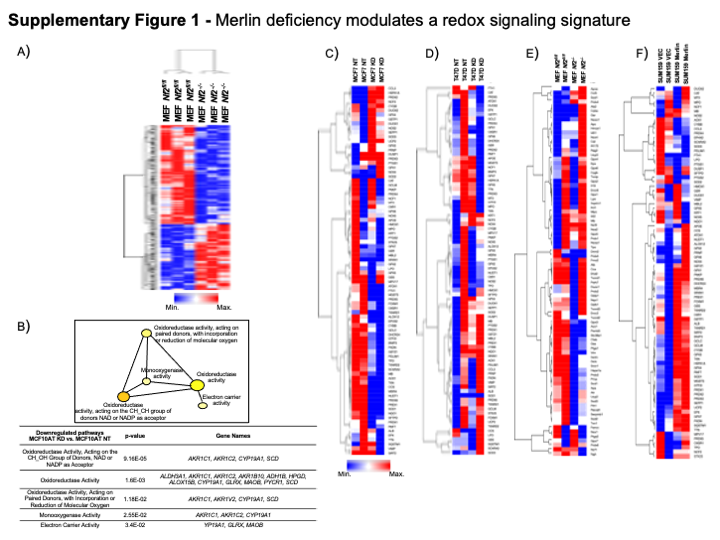

Supplement: Supplementary file 1 — Fig. S1. Merlin deficiency modulates a redox signaling signature. [file MOL2-15-942-s002.tiff]

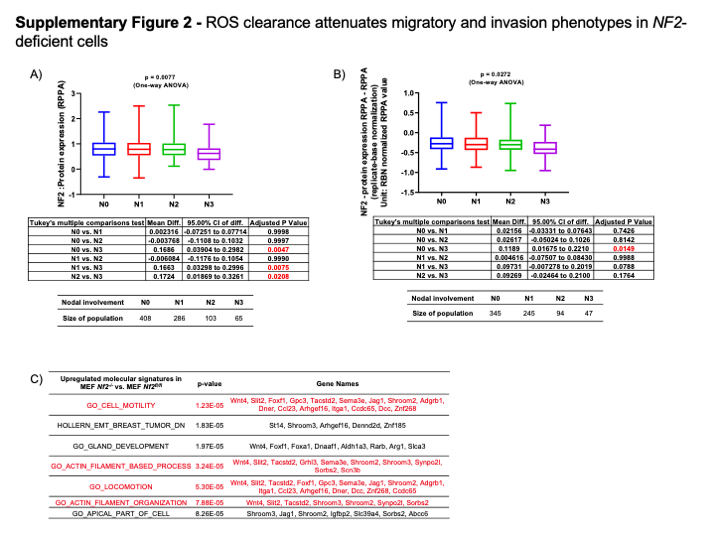

Supplement: Supplementary file 2 — Fig. S2. ROS clearance attenuates migratory and invasion phenotypes in NF2‐deficient cells. [file MOL2-15-942-s003.tiff]

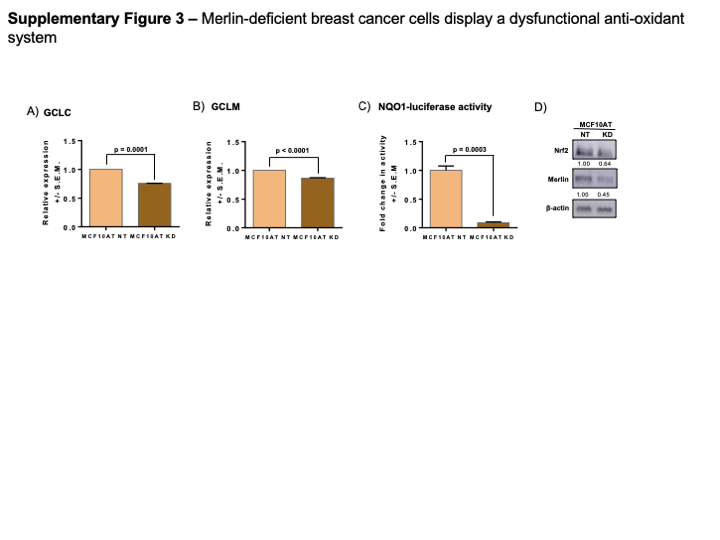

Supplement: Supplementary file 3 — Fig. S3. Merlin‐deficient breast cancer cells display a dysfunctional antioxidant system. [file MOL2-15-942-s005.tiff]

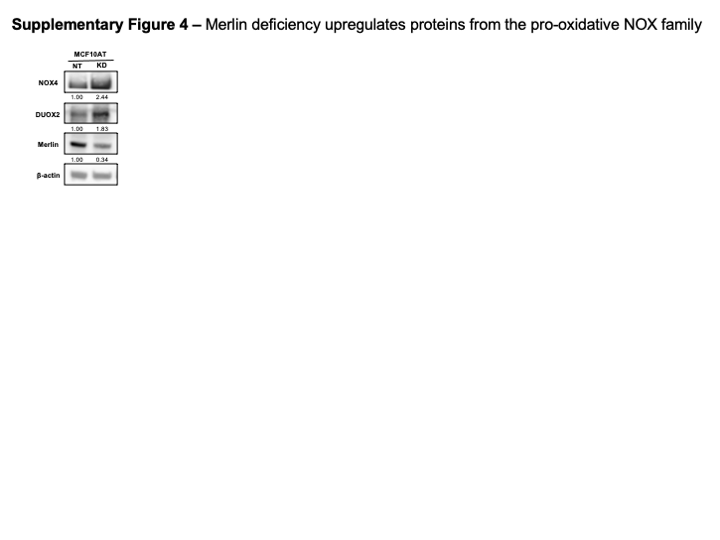

Supplement: Supplementary file 4 — Fig. S4. Merlin deficiency upregulates proteins from the pro‐oxidative NOX family. [file MOL2-15-942-s004.tiff]

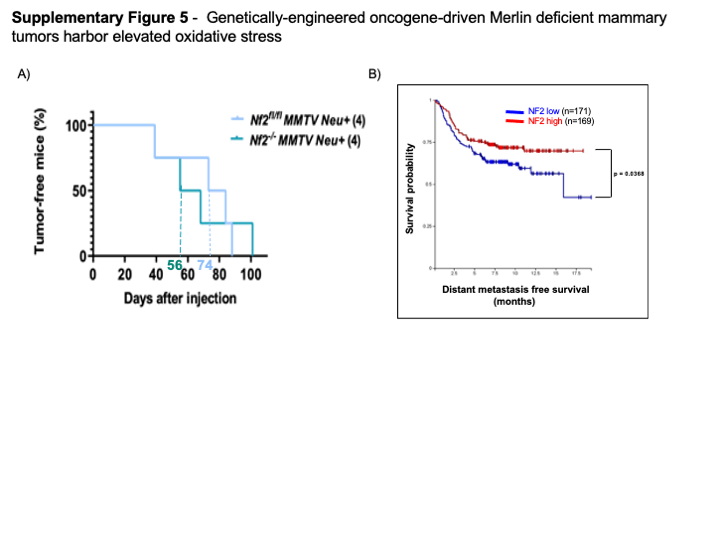

Supplement: Supplementary file 5 — Fig. S5. Genetically engineered oncogene‐driven Merlin‐deficient mammary tumors harbor elevated oxidative stress. [file MOL2-15-942-s001.tiff]
